# Utilisation of the Innovative [18F]-Labelled Radiotracer [18F]-BIBD-071 Within HR+ Breast Cancer Xenograft Mouse Models

**DOI:** 10.3390/ph18010066

**Published:** 2025-01-09

**Authors:** Di Fan, Xin Wang, Xueyuan Ling, Hongbin Li, Lu Zhang, Wei Zheng, Zehui Wu, Lin Ai

**Affiliations:** 1 Department of Nuclear Medicine, Beijing Tiantan Hospital, Capital Medical University, Beijing 100071, China; fandi0929@163.com (D.F.); wangxin920520@yeah.net (X.W.); lhb529@126.com (H.L.); 2 Department of Neurosurgery, Beijing Tiantan Hospital, Capital Medical University, Beijing 100071, China; l15652306297@163.com; 3 Beijing Institute of Brain Disorders, Capital Medical University, Beijing 100069, China; zhanglu970829@outlook.com (L.Z.); zhengwei2019@ccmu.edu.cn (W.Z.)

**Keywords:** [18F]BIBD-071, HR+ breast cancer, aromatase, PET/CT

## Abstract

**Background/Objectives:** Aromatase plays a crucial role in the conversion of androgens to oestrogens and is often overexpressed in hormone-dependent tumours, particularly breast cancer. [18F]BIBD-071, which has excellent binding affinity for aromatase and good pharmacokinetics, has potential for the diagnosis and treatment of aromatase-related diseases. The MCF-7 cell line, which is hormone receptor-positive (HR+), was used in the assessment of the novel [18F]-labelled radiotracer [18F]BIBD-071 via positron emission tomography (PET) imaging of an HR+ breast cancer xenograft model. **Methods:** [18F]BIBD-071 was synthesised, radiolabelled, and then subjected to in vitro stability testing. MCF-7 cells were cultured and implanted into BALB/c nude mice to establish subcutaneous tumour models. MicroPET/CT imaging was conducted after injection of the tracer at 1 and 2 h, and a blocking study was also conducted using the aromatase inhibitor letrozole. A block experiment was used to prove the specificity of the probe. Biodistribution studies were performed at 0.5, 1, and 2 h post injection (p.i.). Immunofluorescence was used to assess aromatase expression in MCF-7 cells. **Results:** [18F]BIBD-071 showed excellent in vitro stability and specific uptake in an MCF-7 xenograft tumour model. MicroPET/CT imaging at 1 and 2 h p.i. revealed excellent tumour visualisation with a favourable tumour-to-background ratio. Biodistribution data revealed high tracer uptake in the liver, small intestine, and stomach, with significant washout from the bloodstream and tumour over time. The tumour uptakes at 0.5 h, 1 h, and 2 h were 3.84 ± 0.13, 2.5 ± 0.17, and 2.54 ± 0.32, respectively. The tumour uptake significantly decreased between 0.5 h and 1 h (*p* < 0.0001), whereas there was no significant difference between 1 and 2 h. The tumour/background ratios at 0.5 h, 1 h, and 2 h were 1.19 ± 0.03, 1.12 ± 0.17, and 1.42 ± 0.11, respectively. Immunofluorescence confirmed robust aromatase expression in MCF-7 cells, which was correlated with [18F]BIBD-071 tumour uptake. **Conclusions:** [18F]BIBD-071 is a promising PET tracer for diagnosing and monitoring HR+ breast cancer, warranting further research into hormone-dependent cancers.

## 1. Introduction

Aromatase is the key enzyme responsible for converting androgens to oestrogens in the body [1]. It is widely expressed in various tissues and organs, such as the central nervous system, placenta, ovaries, testes, mammary glands, bones, liver, uterus, adipose tissue, and skeletal muscle [2,3]. Aromatase is the enzyme responsible for oestrogen biosynthesis, and it is known to be overexpressed in various types of tumours. High aromatase expression has been observed in some of the most common cancers, such as endometrial cancer, prostate cancer, and brain tumours [4,5,6,7,8]. There are also reports of other diseases related to aromatase, such as Alzheimer’s disease, endometriosis, polycystic ovary syndrome, and depression [9,10,11,12].

Breast cancer is the most common cancer among women and a leading cause of cancer death, with an incidence rate of 11.6% [13]. Aromatase is overexpressed in hormone-dependent tumours such as breast and ovarian cancer. This type of cancer is closely associated with hormone levels, particularly in hormone receptor-positive (HR+) breast cancer. In these types of breast cancer, the expression level of aromatase, which is a key enzyme that converts androgens into oestrogens, is often high [7]. The overexpression of aromatase is closely related to the development, progression, and response of breast cancer to treatment. In breast cancer cells, aromatase is highly expressed in the cytoplasm and endoplasmic reticulum and is crucial for the growth of oestrogen-dependent tumours [14]. Oestrogen, as a driving force for the growth of these tumours, directly promotes tumour development via increased synthesis. Therefore, aromatase is not only a key enzyme in oestrogen synthesis but also an important target for the treatment of HR+ breast cancer. The MCF-7 cell line is a human breast cancer cell line that is widely used in breast cancer research, and it is considered a typical representative of cells with an oestrogen receptor-positive status [15]. This cell line has become an important model for studying the pathophysiology of breast cancer and testing new therapies because of its dependence on oestrogen [16].

Positron emission tomography (PET) is a non-invasive imaging technique widely used in clinical settings, particularly for the detection of tumours and neurological diseases [17]. PET imaging using radiolabelled aromatase inhibitors can help visualise aromatase expression in vivo, which may be useful for diagnosing and monitoring HR+ breast cancer [2]. The use of aromatase tracers for positron emission tomography (PET) can be applied to identify highly expressed aromatase tumour locations and diagnose these tumours. During the treatment process, positron emission tomography (PET) can be used to assess the response of highly aromatase-expressing tumours to therapy [3]. Aromatase tracers can not only be used for imaging but also be designed to carry therapeutic radioisotopes as targeted therapeutic agents. These agents can specifically bind to aromatase on tumour cells, releasing radioactive particles that kill or damage tumour cells. The application of aromatase tracers in PET imaging and therapy may become a research field with the potential to improve disease diagnosis and treatment [18].

Small-molecule drugs with aromatic or heteroaromatic systems often require the introduction of radionuclides. We reviewed studies reporting that [11C]-vorozole [2,19,20,21,22] and [11C]-cetrozole [23,24] can function as high-affinity aromatase-binding radiotracers for PET imaging. Owing to the short half-life of [11C], radiopharmaceuticals must be used near the site of production, which limits their delivery and application. In contrast, the longer half-life of [18F] (approximately 110 min) offers a more extended time window for PET imaging. Furthermore, the longer half-life of [18F] allows for the transportation of radioactive pharmaceuticals to more distant locations, enabling a wider range of medical institutions to utilise PET tracers. This is particularly advantageous compared to [11C], which typically requires onsite production. Owing to its superior physical properties, fluorine-18 [18F] has become the predominant radioisotope used in PET imaging [25].

Professor Zehui Wu’s team discovered radiotracers that target aromatase, [18F]BIBD-069 and [18F]BIBD-071, which exhibit excellent binding affinity for aromatase and good pharmacokinetics; as a result, they have potential for the diagnosis and treatment of aromatase-related diseases [26]. [18F]BIBD-071 exhibits favourable pharmacokinetic characteristics, allowing rapid clearance from the body as a diagnostic agent. Compared with [11C]-labelled aromatase tracers, [18F]BIBD-071 provides a longer imaging window, facilitating clinical application and patient scheduling. The radiolabelling conditions are relatively simple, and the yield is high, making it convenient for clinical translation. [18F]BIBD-071 has favourable pharmacokinetic properties, including high bioavailability and an appropriate lipid–water partition coefficient. No defluorination of [18F]BIBD-071 was observed in vivo, which helps to reduce nonspecific signals and improve imaging accuracy. Owing to its superior characteristics, [18F]BIBD-071 has the potential to become an important tool in the clinical diagnosis and treatment of aromatase-related diseases, especially in the monitoring and assessment of oestrogen-dependent diseases [26]. To promote the translation of [18F]BIBD-071 from laboratory research to clinical application and potentially provide new tools for the diagnosis and treatment of aromatase-related diseases, we first applied it to animal models of aromatase-related breast cancer. Moreover, we obtained quantitative biodistribution data of the probe in a breast tumour model through in vivo distribution experiments, which helps us further understand the metabolic behaviour of the radiotracer in important tissues and organs.

In this study, we applied [18F]BIBD-071 in hormone-dependent breast cancer. We plan to conduct further research in other hormone-dependent tumours to explore the diagnostic and therapeutic applications of this aromatase tracer in hormone-dependent diseases. This tracer has the potential to become an important tool for personalised medicine and precision treatment.

## 2. Results

### 2.1. In Vitro Stability of [18F]BIBD-071

**Figure 1:**
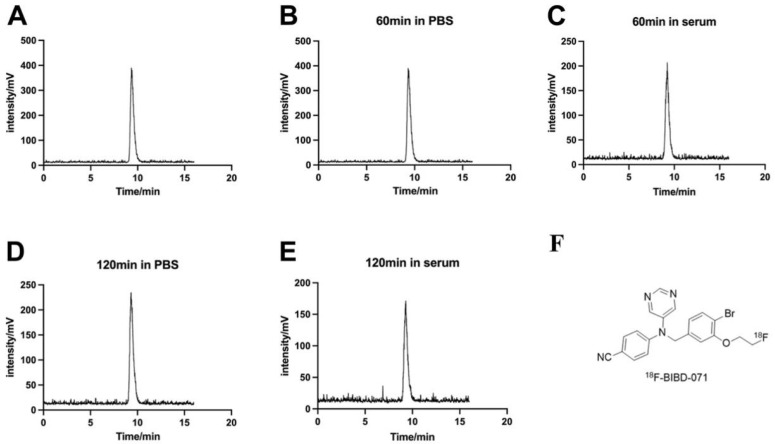
Radioactive HPLC profile of [18F]BIBD-071 (**A**). Radioactive HPLC profile of [18F]BIBD-071 following incubation in PBS or FBS for 60 min (**B**,**C**) and 120 min (**D**,**E**). Structural diagram of [18F]BIBD-071 (**F**).

After radiolabelling, we obtained the probe [18F]BIBD-071, the structure of which is shown in Figure 1F. The radiochemical purity was measured via high-performance liquid chromatography (HPLC), and the radiochemical purity was greater than 98% (A). Radioactive HPLC profile of [18F]BIBD-071 following incubation in PBS or FBS for 60 min (B, C) and 120 min (D, E). The HPLC chromatograms revealed no significant impurities in the samples compared to what was observed at baseline under both conditions (PBS and FBS) after incubation periods of 60 and 120 min. These results indicate that [18F]BIBD-071 is suitable for subsequent experiments.

### 2.2. Micro PET/CT Imaging of the MCF-7 Xenograft Tumour Model

**Figure 2:**
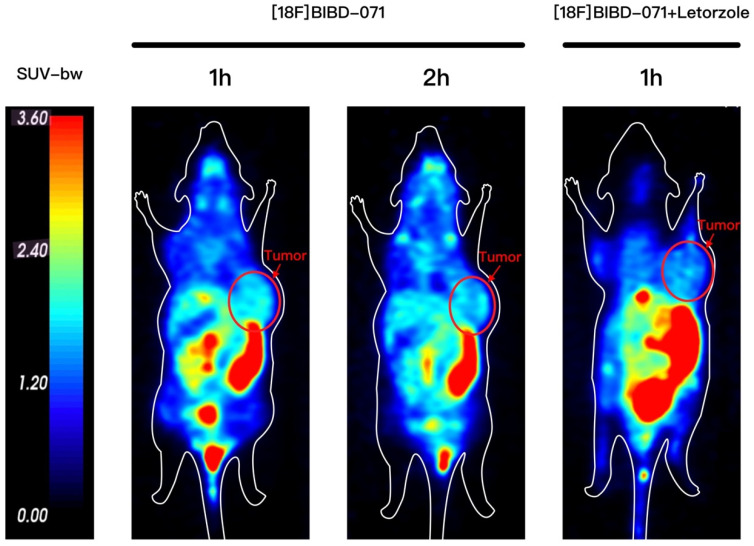
Representative small animal PET images of [18F]BIBD-071 in an MCF-7 xenograft breast tumour model with/without the blocking agent letorzole. The arrows indicate the breast tumour.

In the subcutaneous breast tumour model, small animal PET images revealed that the MCF-7 tumours had a good visual tumour/background ratio at 1 h and 2 h p.i. of [18F]BIBD-071 (Figure 2). The tumour could be observed in the right axillary region, and the contralateral axilla was used as a background for comparison. Through PET/CT imaging, a clear contrast could be visually observed. The tumour uptake of [18F]BIBD-071 was greater at 1 h p.i. than at 2 h p.i. The whole-body background uptake slightly decreased at 2 h p.i., and the tumour could still be clearly visualised. The blockade study for coinjection with the aromatase inhibitor letorzole did not reveal obvious uptake in the tumour area compared with that in the mouse without letorzole, which revealed the specificity of [18F]BIBD-071. The lungs and brain of model mice with low aromatase expression presented low aromatase uptake, and these results are similar to those of previous studies. The strong uptake observed in the abdomen will be further explained in the biodistribution study section.

### 2.3. Biodistribution of [18F]BIBD-071 in a Breast Cancer Xenograft Model

**Figure 3:**
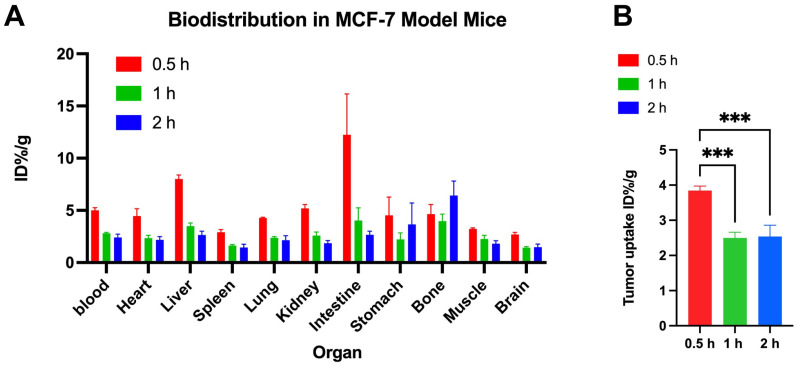
(**A**) Biodistribution of [18F]BIBD-071 in BALB/c nude mice across various organs, with the periods of 0.5, 1, and 2 h as the optimal time frames. (**B**) Tumour uptake at 0.5, 1 and 2 h. (***: *p* < 0.0001) (*n* = 3).

As shown in Figure 3A, [18F]BIBD-071 had high uptake in the intestine and liver and significantly low uptake in the brain and spleen; moreover, there was a more pronounced decline from 0.5 to 1 h in each organ than from 1 to 2 h. At 0.5 h, both blood and tumour uptake were greater than they were at 1 h. The tumour uptake significantly decreased from 0.5 h to 1 h, whereas there was no significant difference between 1 and 2 h, as shown in Figure 3B. The biodistribution in Figure 3A shows high uptake in the liver, small intestine, and stomach, which is consistent with the imaging results. From 0.5 h to 1 h and then to 2 h, the uptake of the probe in nontargeted tissues such as the liver, small intestine, and stomach also significantly decreased. The tumour uptakes at 0.5 h, 1 h, and 2 h were 3.84 ± 0.13, 2.5 ± 0.17, 2.54 ± 0.32, respectively. The tumour/background ratios at 0.5 h, 1 h, and 2 h were 1.19 ± 0.03, 1.12 ± 0.17, and 1.42 ± 0.11, respectively.

### 2.4. Immunofluorescence Analysis of Cells

**Figure 4:**
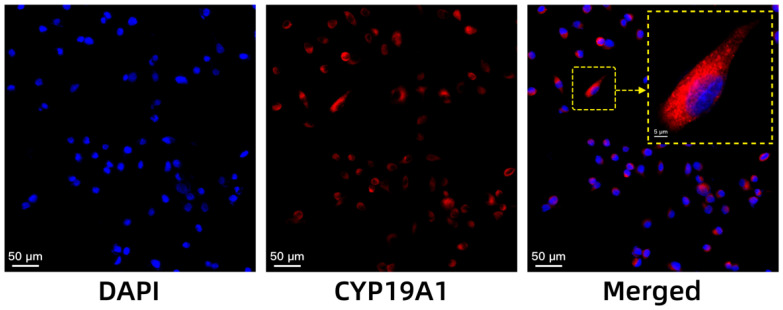
Fluorescence visualisation of the binding between the *CYP19A1* polyclonal antibody and MCF-7 cells, as visualised via ortho-fluorescence microscopy. The scale bar equals 50 µm.

To further determine whether the tumour uptake of [18F]-BIBD-071 is related to the expression level of aromatase, an immunofluorescence analysis was conducted on the cells (Figure 4). Aromatase was highly expressed in the MCF-7 cell line. Immunofluorescence shows specific binding of the *CYP19A1* polyclonal antibody to MCF-7 cells.

## 3. Discussion

In this study, we introduce [18F]-BIBD-071, a novel [18F]-labelled PET tracer specific to aromatase, which was designed and synthesised by Professor Zehui Wu’s team. That original article mentioned two tracers, [18F]-BIBD-069 and [18F]-BIBD-071. We selected [18F]-BIBD-071 for this study because [18F]BIBD-071 exhibits favourable pharmacokinetic characteristics, allowing rapid clearance from the body as a diagnostic agent [26]. Additionally, [18F]BIBD-071 could quickly distribute to the target tissue, and we could observe the uptake of the probe by the tumour at 0.5 h. This tracer had previously only undergone design, synthesis, and biological evaluation and has not been applied in an in vivo animal tumour model.

This study introduces [18F]BIBD-071, a novel PET tracer with a high degree of specificity for visualising aromatase expression, particularly in breast cancer. We conducted our investigation using the widely recognised MCF-7 xenograft model, which is a standard for evaluating the efficacy of new cancer imaging agents. Our research findings demonstrate that [18F]BIBD-071 possesses remarkable in vitro stability, a key factor for reliable PET imaging. Moreover, it shows significant tumour uptake and an advantageous tumour-to-background ratio in PET/CT imaging studies. We injected tumours in the right axillary region and the contralateral axilla as a background for comparison. Through PET/CT imaging, a clear contrast could be visually observed. As shown by the biodistribution results, the uptake of the probe in nontargeted tissues such as the liver, small intestine, and stomach significantly decreased. These attributes are paramount for the precise detection and monitoring of cancerous tissues, which are fundamental components of personalised medicine and precision therapy in oncology. The successful application of [18F]BIBD-071 in the MCF-7 model suggests its potential for clinical translation, offering a promising tool for the management of hormone-dependent breast cancer.

Currently, there are reports on the application of [11C]-labelled aromatase tracers [19,20,21,22,23,24]. However, one of the most significant advantages of [18F]BIBD-071 is that [18F] has a longer half-life than [11C]-labelled tracers do, allowing for a longer imaging window [25]. These advantages could increase the accessibility of PET imaging with this tracer for a broader range of patients and medical centres. When selecting the imaging time points, we referred to the most widely used [18F]-labelled positron diagnostic reagent [18F]FDG (fluorodeoxyglucose) [27]. At present, [18F]FDG imaging in our nuclear medicine is usually performed 40 min to an hour after injection. Therefore, for [18F] BIBD-071, a small-molecule diagnostic probe labelled with [18F], we selected imaging time points of 0.5, 1, and 2 h in our design. We hope that the [18F]-labelled tracers are relatively stable; our study confirmed that [18F]BIBD-071 is sufficiently stable both in PBS and in FBS by using HPLC at the 1 h and 2 h time points, and PET imaging was performed at these intervals. This is what is observed for [18F]-FDG, a commonly used PET imaging tracer, where the optimal imaging time is 40 min to 60 min after the injection of the tracer [27].

In Professor Wu’s article, the biodistribution results revealed greater uptake of the tracer in the stomach, ovaries, and adrenal glands than in other analysed tissues. Our study, however, demonstrated increased uptake in the small intestine, liver, and kidneys. Professor Wu’s experiments were conducted on Sprague–Dawley rats [26], whereas our experiments utilised nude mice. There may be some differences resulting from the different species being used in the studies. Like rats and mice, for the same probe ([18F]BIBD-071), the same organs had varying uptake, and further experiments are needed to explore the distinct organ distributions of this tracer. The biodistribution data revealed high initial blood and tumour uptake, with a subsequent decline, suggesting rapid metabolic clearance. The relatively low uptake in the brain is likely due to the blood–brain barrier and the low expression of aromatase in the brain, which is consistent with the findings of Professor Kayo Takahashi and colleagues [24].

The high expression of aromatase in breast cancer has been well-documented in the literature, highlighting its importance as a therapeutic target [28]. Aromatase, the enzyme responsible for the conversion of androgens to oestrogens, is notably elevated in the cytoplasm and endoplasmic reticulum of breast cancer cells and is crucial for oestrogen-dependent tumour growth [14]. Our immunofluorescence results align with previous findings, confirming that MCF-7 cells, a widely recognised model for HR+ breast cancer, express high levels of *CYP19A1*, the gene encoding aromatase. This high expression validates the choice of the MCF-7 cell line for our experiments, as it provides a relevant and representative model for studying the role of aromatase in breast cancer [15].

Our study has several limitations that cause our findings to warrant further investigation: (1) The high uptake in the liver and small intestine may necessitate further strategies to reduce background signals and enhance tumour visualisation. Subsequent studies should design more imaging time points to find a better balance between a lower background signal and a more suitable tumour signal. Alternatively, adjusting the injection dose may reduce uptake in nontarget organs while maintaining tumour visualisation by reducing the dose. (2) Professor Wu’s team chose Sprague–Dawley rats for drug stability experiments [26], whereas we selected nude mice for the establishment of tumour models. This is because nude mice have an incomplete immune system, which facilitates the establishment of xenograft tumour models. Because different tumour models may lead to different results in biodistribution data, the uptake of biodistribution in the same organs at the same time point may vary. (3) The current study provides valuable insights into the biodistribution and PET imaging of [18F]BIBD-071 in an HR+ breast cancer xenograft model. The next step should be to further investigate its application in other oestrogen-dependent tumour models.

In conclusion, the present study demonstrates the promising potential of the novel [18F]-labelled radiotracer [18F]BIBD-071 in PET imaging for HR+ breast cancer. Our findings revealed that [18F]BIBD-071 exhibited excellent in vitro stability and selective uptake in the MCF-7 xenograft tumour model, with favourable tumour visualisation and a significant tumour-to-background ratio observed via PET/CT imaging at 1 and 2 h postinjection. The biodistribution studies confirmed high tracer uptake in the liver, small intestine, and stomach, with a notable decrease over time. Aromatase expression in MCF-7 cells, as confirmed by immunofluorescence, is correlated with the tumour uptake of [18F]BIBD-071. These results suggest that [18F]BIBD-071 is a valuable PET tracer for diagnosing and monitoring HR+ breast cancer and warrants further investigation into its application in hormone-dependent cancers. Future research should focus on optimising the biodistribution profile of [18F]BIBD-071, exploring its application in other cancer types, and conducting clinical trials.

## 4. Materials and Methods

### 4.1. Synthesis and Radiolabelling of [18F]BIBD-071

[18F]BIBD-071 was synthesised as described previously [26]. The precursor was synthesised by us, and the [18F]-fluoride solution in [18O]H_2_O was purchased from China Beijing HTA Co., Ltd. (Beijing, China). Briefly, an activated Sep-Pak Light QMA Carb was loaded with 740 MBq (20 mCi) of [18F]fluoride and eluted with K2.2.2/K_2_CO_3_. In total, 1 milligram of precursor was dissolved in 1 mL of DMSO and added to [18F] fluoride. The mixture was heated at 80 °C for 10 min and added to water after cooling. The mixture was loaded onto an activated Oasis HLB 3 cm^3^ cartridge, pushed through, and washed with water. The crude product [18F]BIBD-071 was eluted with ethanol, diluted with water, and separated by HPLC with a mobile phase of CH_3_CN/H_2_O (40/60) at a flow rate of 2 mL/min. The fraction containing [18F]BIBD-071 was collected, diluted with water and concentrated using a C18 Sep-Pak cartridge. The product was passed through a sterile membrane filter (0.22 μm) and afforded a formulated solution that was ready for administration.

### 4.2. In Vitro Stability Testing

The quality control of [18F]BIBD-071 used HPLC with a mobile phase of CH_3_CN/H_2_O (40/60) at a flow rate of 2 mL/min. The radiochemical purity was greater than 98%. The stability of [18F]BIBD-071 was assessed over various time points after incubation by conducting radio-HPLC to determine its purity. A total of 0.74 MBq of [18F]BIBD-071 was separately added to 100 μL solutions of phosphate-buffered saline (PBS) or foetal bovine serum (FBS) provided by China Zhejiang Jiangsu Biotechnology Co., Ltd. (Suzhou, China). The radiochemical purity was measured via high-performance liquid chromatography (HPLC) at 60 and 120 min with a mobile phase of CH_3_CN/H_2_O (40/60) at a flow rate of 2 mL/min.

### 4.3. Cell Lines and Culture Conditions

The human breast cancer cell line MCF-7 was purchased from the Institution of Biophysics, Chinese Academy of Sciences. MCF-7 cells were cultured in high-glucose DMEM (GIBCO, Grand Island, NY, USA) supplemented with 10% FBS and 1% penicillin–streptomycin (Procell Life Science & Technology Co., Ltd., Wuhan, China). The cells were grown in a humid incubator at 37 °C with 5% CO_2_.

### 4.4. Animal Models

BALB/c nude mice (female, 4–5 weeks) were purchased from Vital River Laboratory (Animal Technology Co., Ltd., Beijing, China). All animal experiments were performed in accordance with the instructions and permissions of the ethical committee of Beijing Tiantan Hospital (ethics approval number: BNI202404007). Subcutaneous tumour models were established by injecting a suspension of 6 × 10^6^ cells in 100 μL of PBS into the right shoulder of nude mice. The mice were kept in the specific pathogen-free (SPF) degree facility under the following conditions: room temperature maintained at 20 to 26 °C, humidity at 40% to 70%, and cycle of 12 h light and 12 h dark. The average daily feed consumption was 5 g for 8-week-old animals, and the average daily water consumption was 6 to 7 mL for 8-week-old animals. After 2–3 weeks, when the tumour volume reached 200–300 mm^3^, the mice bearing tumours were used for biodistribution and microPET/CT imaging.

### 4.5. Micro PET/CT Imaging

The mice that had been injected with MCF-7 cells were injected with 4.74 MBq (100 μL, 128 μCi) [18F]BIBD-071 via the tail vein. After the tracer injection, the mice were anaesthetised with 2% isoflurane in 1 L/min oxygen and then placed in the prone position for scanning. Representative PET/CT images of mice bearing MCF-7 tumours were collected at 1 and 2 h after intravenous injection of [18F]BIBD-071 via the tail vein. All PET/CT imaging was performed on a dedicated small animal PET/CT scanner (SuperNova^®^, Preclinical, Shanghai, China). The images were reconstructed via the median root prior to correction and were converted to standardised uptake value images. For the blocking experiment, the inhibitor agent letrozole (5 mg/kg, 100 μL per mouse) was injected via the tail vein half an hour before the tracer was injected. Representative PET/CT images of mice bearing MCF-7 tumours were collected at 1 h after intravenous injection of [18F]BIBD-071.

### 4.6. Biodistribution in an MCF-7 Xenograft Tumour Mouse Model

Mice bearing MCF-7 tumours were injected with approximately 0.65 MBq (100 μL, 17.5 μCi) [18F]BIBD-071 via the tail vein. Nine mice were randomly divided into three groups and sacrificed by cervical dislocation at 0.5, 1, and 2 h postinjection (*n* = 3). Organs and tissues of interest, including the blood, heart, liver, spleen, lung, kidney, intestine, stomach, bone, muscle, brain, and tumour, were collected and weighed. The radioactivity of these samples was measured with a gamma counter, and the results are expressed as the percent uptake of the injected dose per gram (%ID/g).

### 4.7. Immunofluorescence for Cells

A total of 2 × 10^5^ cells were seeded onto coverslips that had been placed in 6-well plates. Following overnight culture, the cells were fixed with paraformaldehyde at room temperature and then washed with PBS. The cells were subsequently incubated with the primary antibody overnight in a humid chamber at 4 °C. The following day, the corresponding secondary antibody was added, and the samples were incubated for 50 min at room temperature. Following rinsing three times with PBS, the samples were stained with a DAPI solution to label the nuclei, and the outcome was examined via ortho-fluorescence microscopy.

### 4.8. Statistical Analysis

All the quantitative data are presented as the means ± standard deviations. Statistical tests were performed with GraphPad Prism version 10.0. A *p* value of less than 0.05 was considered statistically significant.

## 5. Conclusions

In conclusion, [18F]BIBD-071 has shown promising results as a PET diagnostic tracer for HR+ breast cancer, which highly expresses aromatase and has excellent in vitro stability and specific tumour uptake. In this study, we first applied it to animal models of aromatase-related breast cancer. We obtained quantitative biodistribution data of the probe in a breast tumour model through in vivo distribution experiments. Future studies should focus on optimising the biodistribution profile, exploring the application of the tracer in other cancer types, and conducting clinical trials to fully realise the potential of [18F]BIBD-071 in the management of breast cancer and potentially other oestrogen-dependent malignancies.

## Data Availability

All the data are presented in the article.

## References

[1] Simpson E.R. (2003). Sources of estrogen and their importance. J. Steroid Biochem. Mol. Biol..

[2] Biegon A. (2016). In vivo visualization of aromatase in animals and humans. Front. Neuroendocrinol..

[3] Molehin D., Filleur S., Pruitt K. (2021). Regulation of aromatase expression: Potential therapeutic insight into breast cancer treatment. Mol. Cell Endocrinol..

[4] Wu R., Liu S., Liu Y., Sun Y., Xiao H., Huang Y., Yang Z., Wu Z. (2020). PET probe with Aggregation Induced Emission characteristics for the specific turn-on of aromatase. Talanta.

[5] Abaffy T., Matsunami H. (2021). 19-hydroxy Steroids in the Aromatase Reaction: Review on Expression and Potential Functions. J. Endocr. Soc..

[6] Gonzalez-Mora A.M., Garcia-Lopez P. (2021). Estrogen Receptors as Molecular Targets of Endocrine Therapy for Glioblastoma. Int. J. Mol. Sci..

[7] Manna P.R., Molehin D., Ahmed A.U. (2016). Dysregulation of Aromatase in Breast, Endometrial, and Ovarian Cancers: An Overview of Therapeutic Strategies. Prog. Mol. Biol. Transl. Sci..

[8] Yague J.G., Lavaque E., Carretero J., Azcoitia I., Garcia-Segura L.M. (2004). Aromatase, the enzyme responsible for estrogen biosynthesis, is expressed by human and rat glioblastomas. Neurosci. Lett..

[9] Prange-Kiel J., Dudzinski D.A., Prols F., Glatzel M., Matschke J., Rune G.M. (2016). Aromatase Expression in the Hippocampus of AD Patients and 5xFAD Mice. Neural Plast..

[10] Garcia-Segura L.M. (2008). Aromatase in the brain: Not just for reproduction anymore. J. Neuroendocrinol..

[11] Koninckx P.R., Fernandes R., Ussia A., Schindler L., Wattiez A., Al-Suwaidi S., Amro B., Al-Maamari B., Hakim Z., Tahlak M. (2021). Pathogenesis Based Diagnosis and Treatment of Endometriosis. Front. Endocrinol..

[12] Azcoitia I., Mendez P., Garcia-Segura L.M. (2021). Aromatase in the Human Brain. Androg. Clin. Res. Ther..

[13] Bray F., Ferlay J., Soerjomataram I., Siegel R.L., Torre L.A., Jemal A. (2018). Global cancer statistics 2018: GLOBOCAN estimates of incidence and mortality worldwide for 36 cancers in 185 countries. CA Cancer J. Clin..

[14] Molehin D., Rasha F., Rahman R.L., Pruitt K. (2021). Regulation of aromatase in cancer. Mol. Cell Biochem..

[15] Lee K., Macaulay V.M., Nicholls J.E., Detre S., Ashworth A., Dowsett M. (1995). An in vivo model of intratumoural aromatase using aromatase-transfected MCF7 human breast cancer cells. Exp. Cancer.

[16] Min D.Y., Jung E., Ahn S.S., Lee Y.H., Lim Y., Shin S.Y. (2020). Chrysoeriol Prevents TNFalpha-Induced *CYP19* Gene Expression via *EGR-1* Downregulation in MCF7 Breast Cancer Cells. Int. J. Mol. Sci..

[17] McConathy J., Goodman M.M. (2008). Non-natural amino acids for tumor imaging using positron emission tomography and single photon emission computed tomography. Cancer Metastasis Rev..

[18] Dave N., Gudelsky G.A., Desai P.B. (2013). The pharmacokinetics of letrozole in brain and brain tumor in rats with orthotopically implanted C6 glioma, assessed using intracerebral microdialysis. Cancer Chemother. Pharmacol..

[19] Biegon A., Shroyer K.R., Franceschi D., Dhawan J., Tahmi M., Pareto D., Bonilla P., Airola K., Cohen J. (2020). Initial Studies with (11)C-Vorozole PET Detect Overexpression of Intratumoral Aromatase in Breast Cancer. J. Nucl. Med..

[20] Biegon A., Alexoff D.L., Kim S.W., Logan J., Pareto D., Schlyer D., Wang G.J., Fowler J.S. (2015). Aromatase imaging with [N-methyl-11C]vorozole PET in healthy men and women. J. Nucl. Med..

[21] Ozawa M., Takahashi K., Akazawa K.H., Takashima T., Nagata H., Doi H., Hosoya T., Wada Y., Cui Y., Kataoka Y. (2011). PET of aromatase in gastric parietal cells using 11C-vorozole. J. Nucl. Med..

[22] Pareto D., Biegon A., Alexoff D., Carter P., Shea C., Muench L., Xu Y., Fowler J.S., Kim S.W., Logan J. (2013). In vivo imaging of brain aromatase in female baboons: [11C]vorozole kinetics and effect of the menstrual cycle. Mol. Imaging.

[23] Takahashi K., Hosoya T., Onoe K., Doi H., Nagata H., Hiramatsu T., Li X.L., Watanabe Y., Wada Y., Takashima T. (2014). 11C-cetrozole: An improved C-11C-methylated PET probe for aromatase imaging in the brain. J. Nucl. Med..

[24] Takahashi K., Hosoya T., Onoe K., Mori T., Tazawa S., Mawatari A., Wada Y., Watanabe Y., Doi H., Watanabe Y. (2021). PET imaging of brain aromatase in humans and rhesus monkeys by (11)C-labeled cetrozole analogs. Sci. Rep..

[25] Erlandsson M., Karimi F., Takahashi K., Långström B. (2008). 18F-Labelled vorozole analogues as PET tracer for aromatase. J. Label. Compd. Radiopharm..

[26] Zheng W., Cheng X., Chen H., Jiang Z., Sun Y., Yu Z., Yang T., Zhang L., Liu Y., Ji X. (2022). Novel (18)F-Labeled PET Tracers Specific to Aromatase: Design, Synthesis, and Biological Evaluation. Mol. Pharm..

[27] Delbeke D., Coleman R.E., Guiberteau M.J., Brown M.L., Royal H.D., Siegel B.A., Townsend D.W., Berland L.L., Parker J.A., Hubner K. (2006). Procedure Guideline for Tumor Imaging with 18F-FDG PET:CT 1.0. J. Nucl. Med..

[28] Harada N. (1997). Aberrant expression of aromatase in breast cancer tissues. J. Steroid Biochem. Mol. Biol..

